# Trends of CD4 cell count levels at the initiation of antiretroviral therapy over time and factors associated with late initiation of antiretroviral therapy among Asian HIV-positive patients

**DOI:** 10.7448/IAS.17.1.18804

**Published:** 2014-03-14

**Authors:** Sasisopin Kiertiburanakul, David Boettiger, Man Po Lee, Sharifah Fs Omar, Junko Tanuma, Oon Tek Ng, Nicolas Durier, Praphan Phanuphak, Rossana Ditangco, Romanee Chaiwarith, Pacharee Kantipong, Christopher Kc Lee, Mahiran Mustafa, Vonthanak Saphonn, Winai Ratanasuwan, Tuti Parwati Merati, Nagalingeswaran Kumarasamy, Wing Wai Wong, Fujie Zhang, Thanh Thuy Pham, Sanjay Pujari, Jun Yong Choi, Evy Yunihastuti, Somnuek Sungkanuparph

**Affiliations:** 1 Faculty of Medicine, Ramathibodi Hospital, Mahidol University, Bangkok, Thailand; 2 The Kirby Institute, University of New South Wales, Kensington, NSW, Australia; 3 Queen Elizabeth Hospital, Hong Kong, China SAR; 4 University of Malaya Medical Centre, Kuala Lumpur, Malaysia; 5 National Center for Global Health and Medicine, Toyama Shinjuku-ku, Tokyo, Japan; 6 Tan Tock Seng Hospital, Singapore; 7 TREAT Asia, amfAR – The Foundation for AIDS Research, Bangkok, Thailand; 8 HIV-NAT/Thai Red Cross AIDS Research Centre, Bangkok, Thailand; 9 Research Institute for Tropical Medicine, Manila, Philippines; 10 Research Institute for Health Sciences, Chiang Mai University, Chiang Mai, Thailand; 11 Chiang Rai Prachanukroh Hospital, Chiang Rai, Thailand; 12 Hospital Sungai Buloh, Sungai Buloh, Malaysia; 13 Hospital Raja Perempuan Zainab II, Kota Bharu, Malaysia; 14 National Center for HIV/AIDS, Dermatology & STDs, Phnom Penh, Cambodia; 15 Faculty of Medicine, Siriraj Hospital, Mahidol University, Bangkok, Thailand; 16 Faculty of Medicine, Udayana University & Sanglah Hospital, Bali, Indonesia; 17 YRG Centre for AIDS Research and Education, Chennai, India; 18 Taipei Veterans General Hospital and AIDS Prevention and Research Centre, National Yang-Ming University, Taipei, Taiwan; 19 Beijing Ditan Hospital, Beijing, China; 20 Bach Mai Hospital, Hanoi, Vietnam; 21 Institute of Infectious Diseases, Pune, India; 22 Division of Infectious Diseases, Department of Internal Medicine, Yonsei University College of Medicine, Seoul, South Korea; 23 Working Group on AIDS Faculty of Medicine, University of Indonesia, Cipto Mangunkusumo Hospital, Jakarta, Indonesia

**Keywords:** AIDS, antiretroviral therapy, Asia, CD4, HIV, trends

## Abstract

**Introduction:**

Although antiretroviral therapy (ART) has been rapidly scaled up in Asia, most HIV-positive patients in the region still present with late-stage HIV disease. We aimed to determine trends of pre-ART CD4 levels over time in Asian HIV-positive patients and to determine factors associated with late ART initiation.

**Methods:**

Data from two regional cohort observational databases were analyzed for trends in median CD4 cell counts at ART initiation and the proportion of late ART initiation (CD4 cell counts <200 cells/mm^3^ or prior AIDS diagnosis). Predictors for late ART initiation and mortality were determined.

**Results:**

A total of 2737 HIV-positive ART-naïve patients from 22 sites in 13 Asian countries and territories were eligible. The overall median (IQR) CD4 cell count at ART initiation was 150 (46–241) cells/mm^3^. Median CD4 cell counts at ART initiation increased over time, from a low point of 115 cells/mm^3^ in 2008 to a peak of 302 cells/mm^3^ after 2011 (*p* for trend 0.002). The proportion of patients with late ART initiation significantly decreased over time from 79.1% before 2007 to 36.3% after 2011 (*p* for trend <0.001). Factors associated with late ART initiation were year of ART initiation (e.g. 2010 vs. before 2007; OR 0.40, 95% CI 0.27–0.59; *p*<0.001), sex (male vs. female; OR 1.51, 95% CI 1.18–1.93; *p*=0.001) and HIV exposure risk (heterosexual vs. homosexual; OR 1.66, 95% CI 1.24–2.23; *p*=0.001 and intravenous drug use vs. homosexual; OR 3.03, 95% CI 1.77–5.21; *p*<0.001). Factors associated with mortality after ART initiation were late ART initiation (HR 2.13, 95% CI 1.19–3.79; *p*=0.010), sex (male vs. female; HR 2.12, 95% CI 1.31–3.43; *p*=0.002), age (≥51 vs. ≤30 years; HR 3.91, 95% CI 2.18–7.04; *p*<0.001) and hepatitis C serostatus (positive vs. negative; HR 2.48, 95% CI 1.−4.36; *p*=0.035).

**Conclusions:**

Median CD4 cell count at ART initiation among Asian patients significantly increases over time but the proportion of patients with late ART initiation is still significant. ART initiation at higher CD4 cell counts remains a challenge. Strategic interventions to increase earlier diagnosis of HIV infection and prompt more rapid linkage to ART must be implemented.

## Introduction

Antiretroviral therapy (ART) has dramatically and consistently reduced HIV-associated morbidity and mortality among patients in both developed and developing countries [1–5]. Early initiation of ART, at higher CD4 cell counts, is one of the predictors of virological success after treatment [6], prevents disease progression and prevents HIV transmission to sexual partners [7]. The European AIDS Clinical Society (EACS) guidelines have recommended ART initiation for patients with AIDS-defining illnesses, patients with HIV-related symptoms and asymptomatic HIV-positive patients with CD4 cell counts <350 cells/mm^3^ [8]. However, the International Antiviral Society (IAS)-USA guidelines and the Department of Health and Human Services (DHHS) guidelines recommend ART initiation in all HIV-positive patients regardless of CD4 cell count [1,9]. Guidelines for resource-limited settings have been revised, increasing the CD4 cell count threshold for ART initiation in asymptomatic HIV-positive patients from 200 to 350 [10] or 500 cells/mm^3^ [11].

At the end of 2011, Asia was home to five million of the estimated 34 million people living with HIV worldwide [2]. According to the Joint United Nations Programme on HIV/AIDS (UNAIDS), approximately eight million people were receiving ART in low- and middle-income countries, which was only 54% of those eligible for ART at the end of 2011 [2]. Under the 2010 World Health Organization (WHO) guidelines, 61% (57%–66%) of all persons eligible for HIV treatment in low- and middle-income countries had obtained ART in 2012. However, under the 2013 WHO guidelines, the 9.7 million people receiving ART in low- and middle-income countries represents only 34% (32%–37%) of the 28.6 (26.5–30.9) million people eligible in 2013. Although many countries in Asia have been able to rapidly scale up access to ART through their national AIDS treatment programmes, most HIV-positive patients are unaware of their HIV serostatus and present in very late stages of disease. For example, a study among newly diagnosed HIV-positive patients in Thailand found that 40% of the patients were unaware of their HIV serostatus, 50% of them presented with clinical AIDS and the median CD4 cell count at diagnosis was 260 cells/mm^3^ [12]. Late diagnosis of HIV infection poses a significant challenge to achieving ART initiation at a threshold greater than 350 cells/mm^3^.

We hypothesized that CD4 cell count levels at ART initiation would increase among HIV-positive patients in Asia in recent years due to the aforementioned changes to the WHO and national guidelines of some countries. In addition, determining factor(s) associated with late ART initiation could identify groups at higher risk. Thus, we aimed to assess CD4 cell count levels at ART initiation over time in treatment-naïve, Asian HIV-positive patients and to determine factors predictive of late ART initiation and associated survival.

## Patients and methods

Our study population consisted of HIV-positive patients enrolled in two regional cohort observational databases, the TREAT Asia HIV Observational Database (TAHOD) and TREAT Asia Studies to Evaluate Resistance-Surveillance (TASER-M). These cohorts have been described previously. Briefly, TAHOD is a prospective multi-centre, observational study of patients with HIV and aims to assess HIV disease natural history in treated and untreated patients in the Asia and Pacific region [13]. Retrospective and prospective data is collected at each site in a sequential sample of patients considered likely to remain in follow-up. Recruitment started in September 2003. TASER-M is a multi-centre, cohort study monitoring the development of HIV drug resistance in patients taking ART [14]. Patients eligible for first- or second-line ART initiation were enrolled sequentially. Data on any previous antiretroviral use was collected retrospectively. Patient recruitment commenced in March 2007 and ceased in 2011. Follow-up data continues to be collected as TASER-M was merged with TAHOD in 2012. The present study included patients naïve to ART (defined as three or more antiretroviral drugs) and with available CD4 cell count and/or an AIDS diagnosis prior to ART initiation. We included the patients who have been enrolled in the cohorts since September 2003 and date of data censoring was 31 March 2013.

Study endpoints were CD4 cell count at ART initiation, median CD4 cell count at ART initiation trends by year, late ART initiation defined as CD4 cell count <200 cells/mm^3^ or having a clinical AIDS diagnosis prior to ART initiation and mortality. Our definition of prior AIDS was the diagnosis of the Centers for Disease Control and Prevention (CDC) category C prior to ART initiation. Ethics approvals were obtained from institutional review boards at each of the participating clinical sites where study patient enrolment took place, as well as by separate review boards for the coordinating centre (Therapeutics Research, Education, and AIDS Training in [TREAT] Asia, Bangkok) and the data management and analysis centre (The Kirby Institute, University of New South Wales, Sydney). All patients have their data stored in both the site-level and centralized study databases for the purposes of research.

### Statistical analysis

Baseline values were compared using the Wilcoxon-Mann-Whitney test for continuous variables or Chi-squared test for categorical variables. Trends in median CD4 cell count at ART initiation by year and the proportion of late ART initiation by year were evaluated using Pearson's correlation coefficient and Chi-squared methods, respectively. Data were stratified where permitted by sufficient patient numbers. Predictors associated with late ART initiation were analyzed by logistic regression, conditional upon study site. Predictors of mortality were analyzed using Kaplan-Meier survival estimations and Cox survival analyses, stratified by study site. Predictors included in the multivariate model were selected based on a significance level of ≤0.1 in the univariate analyses. Predictors were retained in the multivariate model if one or more categories exhibited a *p-*value ≤0.05. Stata statistical software version 12.1 was used for all statistical analyses.

## Results

A total of 2737 HIV-positive ART-naïve patients from 22 sites in 13 Asian countries and territories were included in the analysis. Baseline characteristics of all patients stratified by late and non-late ART initiation are shown in Table 1. Approximately 70% were male, 43% were aged 31–40 years and 63% were exposed to HIV via heterosexual contact. Half of the patients had prior AIDS diagnosis. Nearly a quarter (23.9%) of patients initiated ART in 2007 or earlier and 29% of patients initiated ART in 2010 and after. The distributions of HIV exposure category, year at ART initiation, levels of HIV RNA, baseline ART regimen and country income status were significantly different among patients with late ART initiation compared to those with non-late ART initiation (*p*<0.001 for all).

**Table 1:** Patient baseline characteristics

Characteristics	All patients (*n*=2737)	Patients with late ART initiation (*n*=2074)	Patients with non-late ART initiation (*n*=663)	p**
Age (years), *n* (%)				0.038
≤30	610 (22.3)	443 (21.4)	167 (25.2)	
31–40	1166 (42.6)	882 (42.5)	284 (42.8)	
41–50	662 (24.2)	526 (25.4)	136 (20.5)	
≥51	299 (10.9)	23 (10.8)	76 (11.5)	
Sex, *n* (%)				0.769
Male	1903 (69.5)	1439 (69.4)	464 (70.0)	
Female	834 (30.5)	635 (30.6)	199 (20.0)	
HIV exposure category, *n* (%)				<0.001
Heterosexual sex	1732 (63.3)	1377 (66.4)	355 (53.5)	
Homosexual sex	634 (23.2)	403 (19.4)	231 (34.8)	
Intravenous drug use	185 (6.8)	162 (7.8)	23 (3.5)	
Other*	186 (6.8)	132 (6.4)	54 (8.1)	
HBV status, *n* (%)				0.442
Positive	244 (8.9)	188 (9.1)	56 (8.5)	
Negative	1966 (71.8)	1477 (71.2)	489 (73.8)	
Unknown	527 (19.3)	409 (19.7)	118 (17.8)	
HCV status, *n* (%)				0.078
Positive	212 (7.8)	170 (8.2)	42 (6.3)	
Negative	1764 (64.5)	1314 (63.4)	450 (67.9)	
Unknown	761 (27.8)	590 (28.5)	171 (25.8)	
Prior AIDS diagnosis, *n* (%)	1427 (52.1)	1427 (68.8)	0 (0.0)	NA
Year of ART initiation, *n* (%)				<0.001
Before 2007	422 (15.4)	334 (16.1)	88 (13.3)	
2007	233 (8.5)	192 (9.3)	41 (6.2)	
2008	624 (22.8)	510 (24.6)	114 (17.2)	
2009	671 (24.5)	526 (25.4)	145 (21.9)	
2010	579 (21.2)	418 (20.2)	161 (24.3)	
2011	117 (4.3)	61 (2.9)	56 (8.5)	
After 2011	91 (3.3)	33 (1.6)	58 (8.8)	
Median (IQR) CD4 cell count at ART initiation, cells/mm^3^***	150 (46–241)	92 (31–171)	278 (236–329)	<0.001
HIV RNA (copies/mL), *n* (%)				<0.001
≤ 20,000	399 (14.6)	222 (10.7)	177 (26.7)	
20,001–100,000	672 (24.6)	493 (23.8)	179 (27.0)	
100,001–250,000	508 (18.6)	428 (20.6)	80 (12.1)	
≥250,001	529 (19.3)	456 (22.0)	73 (11.0)	
Unknown	629 (23.0)	475 (22.9)	154 (23.2)	
Baseline ART regimen, *n* (%)				<0.001
NRTI plus NNRTI	2446 (89.4)	1885 (90.9)	561 (84.6)	
NRTI and/or NNRTI plus PI	258 (9.4)	162 (7.8)	96 (14.5)	
NRTI only	33 (1.2)	27 (1.3)	6 (0.9)	
Country income level, *n* (%)				
Low/low-middle	663 (24.2)	482 (23.2)	181 (27.3)	
Upper middle	1590 (58.1)	1281 (61.8)	309 (46.6)	<0.001
High	484 (17.7)	311 (15.0)	173 (26.1)	

* Includes those exposed to blood products and unknown exposures;

** *p-*values refer to differences between groups with late and non-late ART initiation

*** overall numbers of patients with baseline CD4 cell count were 2557. Numbers of patients with late ART initiation with CD4 cell count were 1894 and those with non-late ART initiation with CD4 cell count were 663; ART, antiretroviral therapy; HBV, hepatitis B virus; HCV, hepatitis C virus; NRTI, nucleoside reverse transcriptase inhibitor; NNRTI, non-nucleoside reverse transcriptase inhibitor; PI, protease inhibitor.

**Figure 1:**
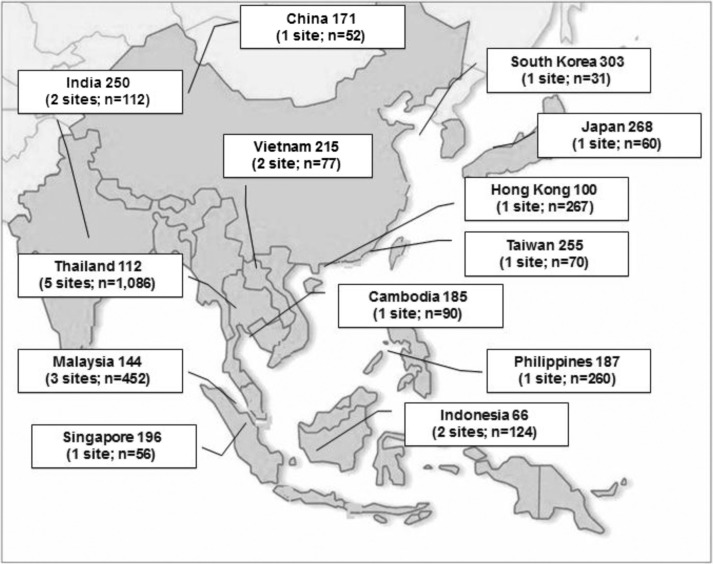
Median CD4 cell counts (cells/mm^3^) at antiretroviral therapy initiation and number of patients from 22 study sites in 13 Asian countries and territories.

The overall median (interquartile range, IQR) CD4 cell count at ART initiation was 150 (46–241) cells/mm^3^. A total of 2074 (76%) patients were classified as having late ART initiation. Median CD4 cell count was 92 (31–171) cells/mm^3^ among patients with late ART initiation and 278 (236–329) cells/mm^3^ among patients with non-late ART initiation (*p*<0.001). By World Bank income status, median CD4 cell count at ART initiation was 186 (75–274), 122 (40–216) and 187 (56–284) cells/mm^3^ in low/lower middle (*n*=592), upper middle (*n*=1492) and high-income (*n*=473) countries, respectively ( *p*<0.001). Median CD4 cell counts at ART initiation by country are shown in Figure 1.

**Figure 2:**
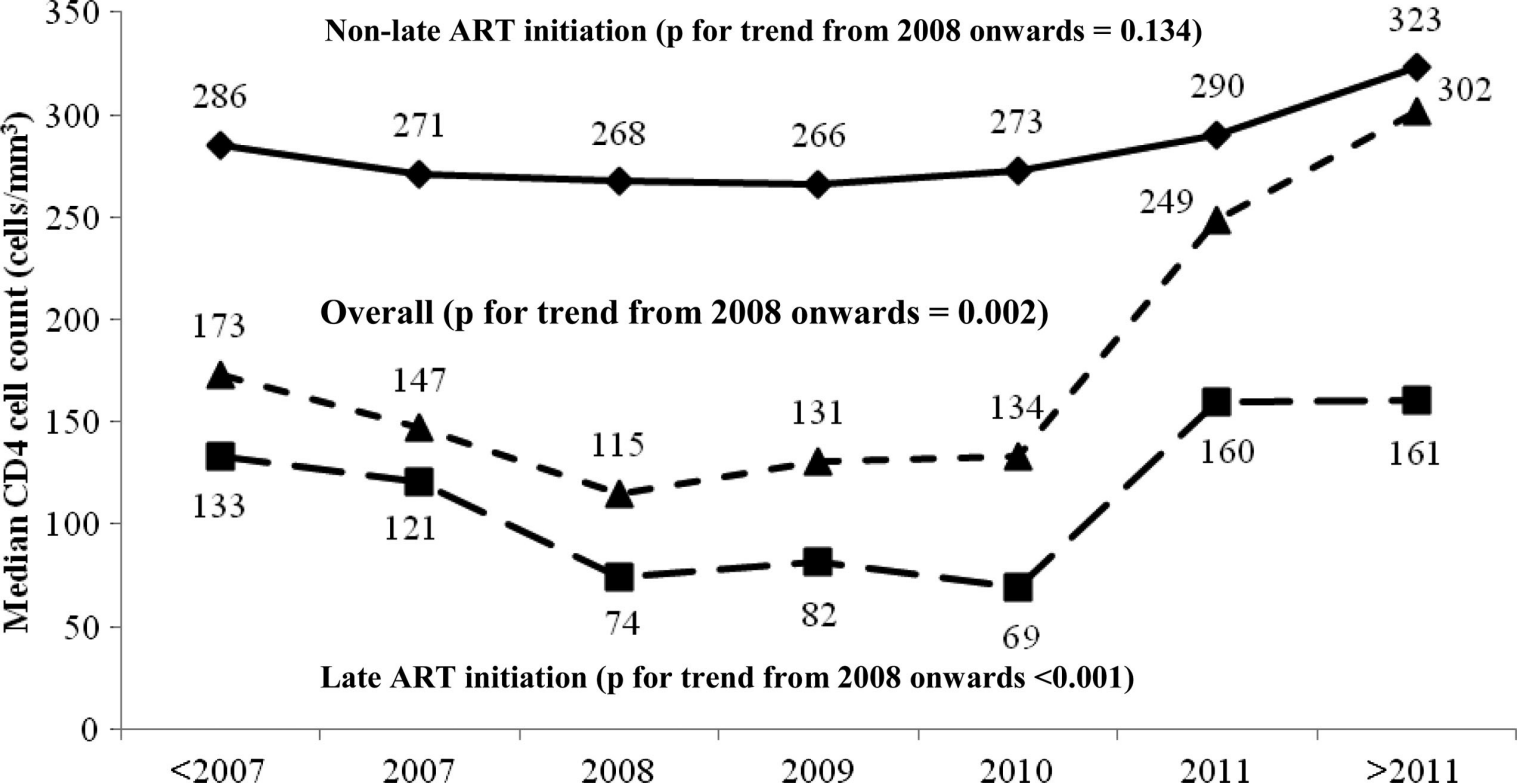
Median CD4 cell count at antiretroviral therapy initiation by calendar year (overall; *n*=2557) stratified by timing of antiretroviral therapy initiation (late initiation; *n*=1894 vs. non-late initiation; *n*=663).

Trends in median CD4 cell count at ART initiation were evaluated from 2008 onwards. Overall CD4 cell count at ART initiation increased from a low point of 115 cells/mm^3^ in 2008 to a peak of 302 cells/mm^3^ for patients starting ART after 2011 (*p* for trend 0.002) (Figure 2). In patients with late ART initiation, CD4 cell count increased from 74 cells/mm^3^ to 161 cells/mm^3^ (*p* for trend <0.001). Of overall CD4 cell count at ART initiation, by sex, median CD4 cell count increased from 115 cells/mm^3^ to 299 cells/mm^3^ in males (*p* for trend 0.009); and from 115 cells/mm^3^ to 309 cells/mm^3^ in females with no significant change (*p* for trend 0.119). By HIV exposure group, median CD4 cell count increased from 100 cells/mm^3^ to 326 cells/mm^3^ in the heterosexual risk group (*p* for trend 0.002) and from 136 cells/mm^3^ to 270 cells/mm^3^ in the homosexual risk group with no significant change (*p* for trend 0.218).

**Figure 3:**
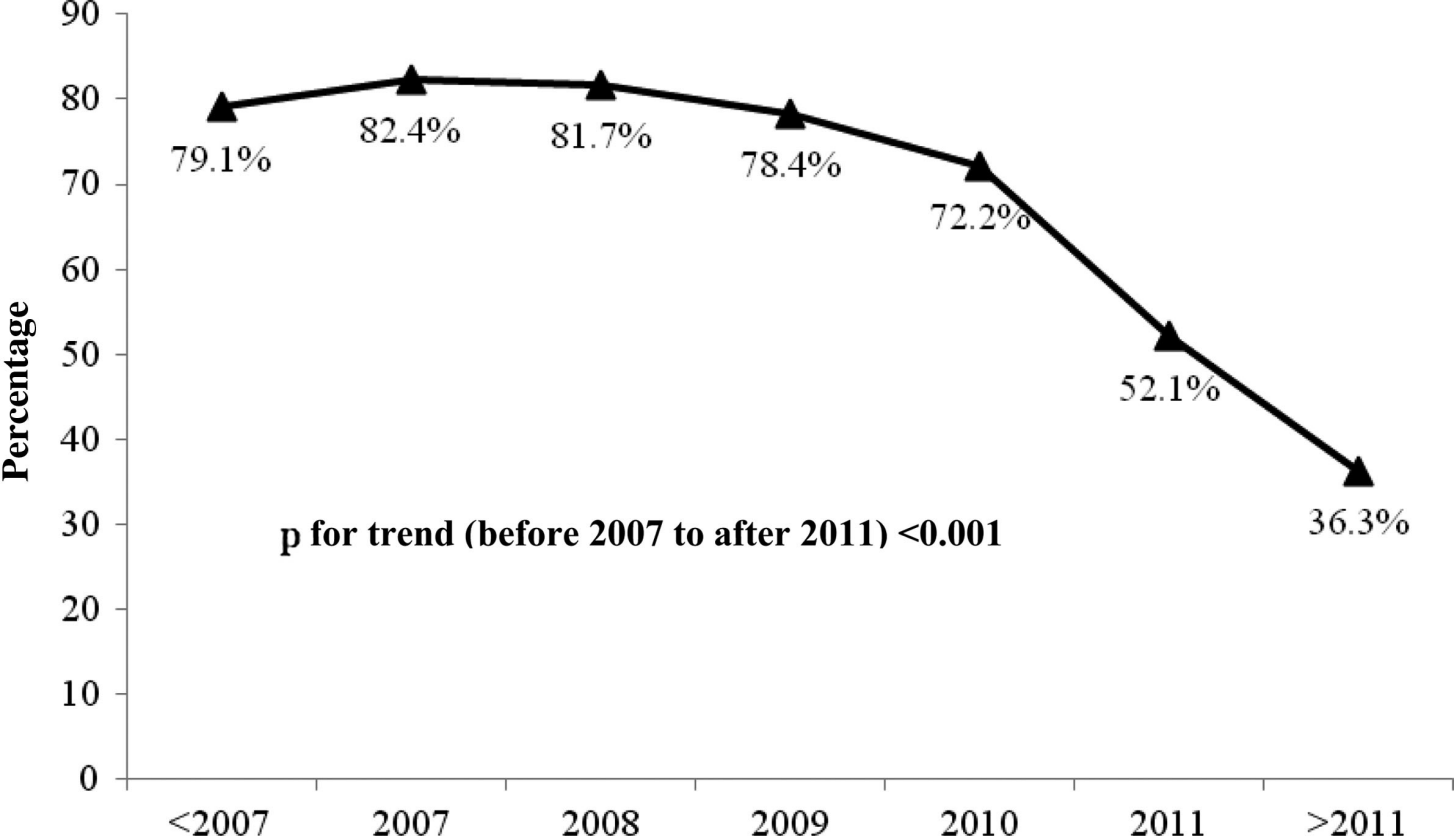
Proportion of patients with late antiretroviral therapy initiation by calendar year.

The proportion of patients with late ART initiation significantly decreased over time from 79.1% before 2007 to 36.3% after 2011 (*p* for trend <0.001) (Figure 3). By sex, the proportion of patients with late ART initiation decreased from 75.9% to 34.6% in male (*p* for trend <0.001) and from 86.4% to 38.5% in females (*p* for trend <0.001). If late ART initiation was defined only as ART initiation at CD4 cell count <200 cells/mm^3^, there was statistically significant decrease in the proportion of those with late ART initiation over time from 71.2% to 20.9% in all patients, from 69.3% to 25.0% in males and from 75.9% to 15.4% in females (*p* for trend from 2008 to after 2011 <0.001 for all). Among late starters, the proportion of patients with prior AIDS at ART initiaition decreased from 79.9% to 62.3% in all patients, from 80.9% to 67.4% in males and from 78.1% to 50.0% in females (*p* for trend before 2007–2011<0.001, all).

By multivariate analysis, year of ART initiation was associated with late ART initiation. Patients starting ART in later years of the study period were less likely to be late starters. This association strengthened over time and became significant from 2010 onwards when compared with those starting ART before 2007. Other predictors associated with late ART initiation were sex (male vs. female; OR 1.51, 95% CI 1.18–1.93; *p*<0.001) and HIV exposure (heterosexual vs. homosexual; OR 1.66, 95% CI 1.24–2.23; *p*=0.001 and intravenous drug use vs. homosexual; OR 3.03, 95% CI 1.77–5.21; *p*<0.001) (Table 2).

**Table 2:** Predictors associated with late antiretroviral therapy initiation

Variables*	Univariate odds ratio (95% CI)	*p*	Multivariate odds ratio (95% CI)	*p*
Year of ART initiation				
Before 2007	reference		reference	
2007	0.97 (0.62–1.52)	0.885	1.00 (0.63–1.57)	0.993
2008	1.01 (0.70–1.46)	0.951	1.03 (0.71–1.49)	0.887
2009	0.69 (0.49–0.99)	0.043	0.72 (0.50–1.03)	0.070
2010	0.33 (0.21–0.50)	<0.001	0.35 (0.23–0.54)	<0.001
2011	0.29 (0.17–0.49)	<0.001	0.32 (0.19–0.56)	<0.001
After 2011	0.13 (0.07–0.23)	<0.001	0.15 (0.08–0.27)	<0.001
Sex				
Female	reference		reference	
Male	1.41 (1.13–1.75)	0.002	1.51 (1.18–1.93)	0.001
HIV exposure category				
Homosexual sex	reference		reference	
Heterosexual sex	1.55 (1.19–2.02)	0.001	1.66 (1.24–2.23)	0.001
Intravenous drug use	3.58 (2.09–6.14)	<0.001	3.03 (1.77–5.21)	<0.001
Other	1.31 (0.88–1.95)	0.180	1.32 (0.88–1.99)	0.180

* Exposure category “other” includes those exposed to blood products and unknown exposures; exclude variables after univariate analyses were age category, HBV status, HCV status, and ART category. Other covariates remained significant in the multivariate model; ART, antiretroviral therapy; CI, confidence interval.

Of 136 (5.0% of total) patients who died, 122 (4.5%) were late ART initiators and 14 (0.5%) were non-late ART initiators. By multivariate Cox proportional hazard regression, late ART initiation was associated with mortality [hazard ratio (HR) 2.13, 95% CI 1.19–3.79; *p*=0.010]. Predictors associated with mortality after ART initiation included sex (male vs. female; HR 2.12, 95% CI 1.31–3.43; *p*=0.002), age (≥51 vs. ≤30 years; HR 3.91, 95% CI 2.18–7.04; *p*<0.001) and hepatitis C virus (HCV) serostatus (positive vs. negative; HR 2.48, 95% CI 1.48–4.16; *p*=0.001) (Table 3).

**Table 3:** Predictors associated with mortality after antiretroviral therapy initiation among 2737 patients

Variables	Univariate hazard ratio (95% CI)	*p*	Multivariate hazard ratio (95% CI)	*p*
Late ART initiation status				
CD4 cell count >200 cells/mm^3^ and no prior AIDS	reference	0.004	reference	0.010
CD4 cell count <200 cells/mm^3^ or had prior AIDS	2.35 (1.31–4.19)		2.13 (1.19–3.79)	
Sex				
Female	reference		reference	
Male	2.55 (1.58–4.11)	<0.001	2.21 (1.31–3.43)	0.002
Age at ART initiation, years				
≤30	reference		reference	
31–40	1.10 (0.63–1.91)	0.738	1.04 (0.60–1.80)	0.896
41–50	1.81 (1.03–3.21)	0.041	1.74 (0.98–3.08)	0.060
≥51	4.02 (2.24–7.22)	<0.001	3.91 (2.18–7.04)	<0.001
HCV status at ART initiation				
Negative	reference		reference	
Positive	2.50 (1.50–4.18)	<0.001	2.48 (1.48–4.16)	0.001

Excluded variables after univariate analyses were HBV status and ART category. Exposure category and year of ART initiation were excluded after multivariate analysis; ART, antiretroviral therapy; CI, confidence interval; HCV, hepatitis C virus.

## Discussion

This is one of the largest studies regarding CD4 cell count levels over time at ART initiation among Asian HIV-positive patients. The overall median CD4 cell count at ART initiation in this study was 150 cells/mm^3^. This is comparable to other studies conducted in Asia, South America and sub-Saharan Africa, which ranged from 67 to 234 cells/mm^3^ [15–19]. Unexpectedly, patients in low/lower middle-income countries had higher median CD4 cell count levels at ART initiation compared to the upper middle-income categories and comparable to those of patients in high-income countries. However, patients in all categories of country income in the present study had a median CD4 cell count <200 cells/mm^3^. Our results contrast with the study by Braitstein *et al*. reporting that patients starting ART in low-income settings had lower CD4 cell counts [17]. Country income may not be an optimal surrogate marker of access to ART, as local policy commitment and stigma are as or even more important than the strength of the national economy.

Median CD4 cell count in our cohort increased over time, especially beyond 2010, which was the time when the WHO had revised the recommendation on CD4 cell count threshold for ART initiation [11]. However, most of the patients had CD4 cell counts <350 cells/mm^3^, which indicated that patients in our cohorts continued entering care late. Based on a meta-regression among 169,007 patients in 44 studies from the United States and Europe, mean CD4 cell count at presentation to care increased minimally by 1.5 cells/mm^3^ per year, from 307 cells/mm^3^ in 1992 to 336 cells/mm^3^ in 2011 [20]. The proportion of our patients with late ART initiation had significantly decreased over time; however a significant number of the patients had prior AIDS diagnosis before ART initiation. A study conducted in Mozambique reported a similar trend, the proportion of patients with late ART initiation decreased from 46% in 2005 to 37% in 2007, but remained constant from 2007 to 2009 [21]. Late ART initiation in the study in Mozambique was defined as CD4 cell count <100 cells/mm^3^ or WHO stage IV.

In this study, several factors were found to be associated with late ART initiation; earlier year of ART initiation, male sex, and heterosexual sex and intravenous drug use as the primary reported HIV exposure category. In the years leading up to and since the revised WHO guidelines, altered health care practices may have led to reductions in the number of patients with late ART initiation over time, but these interventions have not consistently reached those targeted population. The expansion of HIV testing for pregnant women in antenatal care through national prevention of mother to child transmission (PMTCT) programmes led to earlier identification of HIV infection among asymptomatic women in many Asian countries such as Thailand [22]. This is consistent with data from sub-Saharan Africa, where PMTCT coverage was even higher than that in Asia and women tend to initiate ART at an earlier stage of HIV infection than men, although PMTCT coverage remains consistently higher than in Asia [23,24]. These factors may partly explain why male sex was associated with late ART initiation. UNAIDS continues to report low HIV testing rates among intravenous drug users. Among 57 countries reporting, a median of 39% (22%–60%) of people who inject drugs reached in surveys in capital cities reported having received an HIV test in the previous 12 months [2]. In addition, intravenous drug users may be excluded from HIV services because of stigma, discrimination and criminalization. Factors associated with a lower likelihood of late ART initiation such as being female, younger age, marital status and higher education level were reported in a Mozambique cohort [21].

Late ART initiation was statistically associated with mortality in our cohort. Other predictors for mortality were male sex, older age, high baseline HIV RNA and positive HCV serostatus at baseline. Some studies have reported sex differences in mortality as well as CD4 cell count responses to ART. In research on HIV treatment outcomes in Tanzania and South Africa, females had a lower risk of death and made larger CD4 cell count gains after ART initiation [25,26]. ART initiation at an older age is associated with a less robust CD4 cell count response, and starting ART at a younger age may result in better immunologic and clinical outcomes [27–29].

HIV infection is associated with more rapid progression of viral hepatitis-related liver disease, including cirrhosis, end-stage liver disease, hepatocellular carcinoma and fatal hepatic failure [1]. Earlier ART in individuals co-infected with HCV is associated with slower progression of hepatic fibrosis and reduced risk of liver disease progression [30,31]. Our results showed that patients with HCV co-infection had a higher mortality compared to those who had only HIV infection. Treatment of HCV infection is still largely inaccessible in Asia, especially in the low- and middle-income countries. Unfortunately, information on the treatment of HCV infection could not be retrieved from our database, but is expected to be rare in co-infected patients outside of high-income settings.

Our results may not be directly generalizable across the Asia region. These data are from referral centres where patient acuity levels may not be representative of their respective national HIV programmes. We have described our data as being in Asian patients, but recognize the intra-regional and inter-ethnic variations across the participating study sites. Looking at change of CD4 cell count at ART initiation with time within each national ART programme may be a better way to determine programme success in getting patients treated earlier. Furthermore, the total number of patients included in the analysis is relatively small and reflects subsets of individual site-level cohorts. Due to the observational nature of the database, it is unclear if the late ART initiation in our study was the result of late HIV diagnosis, delayed enrolment into HIV care, or late ART initiation despite timely enrolment in care. By using a combined definition of late ART initiation that included prior AIDS diagnoses, we were able to include patients who did not have available CD4 tests at ART initiation into the analysis. However, this makes it more difficult to compare our results to other studies that used solely pre-ART CD4 cell counts to categorize patients.

In conclusion, the median CD4 cell count at ART initiation was low in our cohort, however did increase over time. Although the proportion of those with late ART initiation decreased, a significant number of patients were still late to receive ART. Earlier ART initiation at higher CD4 cell counts remains a challenge. Strategic interventions to increase earlier diagnosis of HIV infection, linkage to HIV care and prompt more rapid access to ART must be implemented in Asian countries, especially among key populations with poor access to HIV testing services.
